# Advancement of magma fragmentation by inhomogeneous bubble distribution

**DOI:** 10.1038/s41598-017-16941-x

**Published:** 2017-12-01

**Authors:** M. Kameda, M. Ichihara, S. Maruyama, N. Kurokawa, Y. Aoki, S. Okumura, K. Uesugi

**Affiliations:** 1 Department of Mechanical Systems Engineering, Tokyo University of Agriculture and Technology, Koganei, Tokyo 184-8588 Japan; 2 Earthquake Research Institute, University of Tokyo, Bunkyo-ku, Tokyo 113-0032 Japan; 3 Department of Earth Science, Tohoku University, Sendai, Miyagi 980-8578 Japan; 4 Japan Synchrotron Radiation Research Institute, Sayo-cho, Hyogo 679-5198 Japan

## Abstract

Decompression times reported in previous studies suggest that thoroughly brittle fragmentation is unlikely in actual explosive volcanic eruptions. What occurs in practice is *brittle-like fragmentation*, which is defined as the solid-like fracture of a material whose bulk rheological properties are close to those of a fluid. Through laboratory experiments and numerical simulation, the link between the inhomogeneous structure of bubbles and the development of cracks that may lead to brittle-like fragmentation was clearly demonstrated here. A rapid decompression test was conducted to simulate the fragmentation of a specimen whose pore morphology was revealed by X-ray microtomography. The dynamic response during decompression was observed by high-speed photography. Large variation was observed in the responses of the specimens even among specimens with equal bulk rheological properties. The stress fields of the specimens under decompression computed by finite element analysis shows that the presence of satellite bubbles beneath a large bubble induced the stress concentration. On the basis of the obtained results, a new mechanism for brittle-like fragmentation is proposed. In the proposed scenario, the second nucleation of bubbles near the fragmentation surface is an essential process for the advancement of fragmentation in an upward magma flow in a volcanic conduit.

## Introduction

The rapid decompression of vesicular magma is an important mechanism in brittle fragmentation^1–3^, which leads to explosive volcanic eruptions^4,5^. Factors controlling the fragmentation process have been investigated through laboratory experiments using shock tube apparatuses with natural volcanic rocks^3,6^, porous solids^7^, synthetic magma^8^, and viscoelastic or viscous fluids^2,9–13^. The fragmentation of solid samples in such experiments occurs when the decompression amplitude is large, the void fraction is large, and the permeability is small^3,6,14^. The results have led to fragmentation criteria based on the critical stress fracture criteria for brittle failure. Different models use different stresses to define the critical stress, including the bulk stress of the porous material^15,16^ and the stress around bubbles with overpressure^17,18^. Recently, Heap *et al*.^19^ demonstrated by numerical calculation the importance of a small external differential stress in addition to the bubble overpressure and the inhomogeneous distribution of bubbles on the initiation and progression of fragmentation.

The fragmentation criterion not only indicates the conditions under which fragmentation occurs but also defines the fragmentation speed. Fragmentation experiments have revealed that the fragmentation front proceeds into the sample at a certain speed depending on the overpressure and void fraction^6,17^. Alidibirov^17^ derived a formula for the fragmentation speed by combining the fragmentation criterion with the equations for the conservation of mass and energy across the fragmentation surface. In his derivation, the conservation of momentum equation is replaced by an assumed power-law relationship between the pressure and the density, considering that rate of momentum transfer depends on the process of gas liberation from disrupted pores and is not known. Koyaguchi & Mitani^20^ eliminated this problem by assuming fragmentation is instantaneous after the critical stress has been reached. Koyaguchi *et al*.^21^ have demonstrated that their model can fit the experimentally observed fragmentation speed^6^ if the stress at the midpoint of the bubble wall is used as the measure of critical stress. More recent theoretical models^16,22^ have revealed that the experimentally observed speed and layer-by-layer behavior of fragmentation is generated by the combined effect of the sample adhering to the shock tube and the escape of permeable gas.

Because magma is a viscoelastic fluid, whether it meets the fragmentation criterion for solid should depend on the rate and time of the process^23,24^. Decompression experiments using viscoelastic fluids have shown that their fragmentation and expansion behaviors are controlled by a combination of three characteristic times: the decompression time *t*_dec_, the relaxation time *τ*_r_ of the material, and the viscous expansion time *τ*_v_ of the bubbles^2,12,25^. If *t*_dec_ is less than *τ*_r_, solid-like brittle fragmentation occurs. If *t*_dec_ is greater than *τ*_*v*_, the bubbles simply expand without fragmentation. Kameda *et al*.^12^ found that when *τ*_r_ < *t*_dec_ < *τ*_v_, solid-like fragmentation occurs but with a significant time delay. They defined this type of fragmentation behavior as *brittle-like fragmentation*. From their detailed observation, Kameda *et al*.^13^ found that the onset of brittle-like fragmentation is triggered by the sudden release of a considerable amount of gas from a crack on the specimen surface. They suggested that the crack initiates in the interior of the specimen by the ductile rupturing of the continuous phase or connection of the bubbles as a result of the complicated morphology of the bubble distribution.

Considering brittle-like fragmentation is particularly important in modeling volcanic eruptions for the following reasons. First, comparing estimations of *t*_dec_ in real explosive events^26–28^ with the value of *τ*_r_ for the silicate melt^29,30^ indicates that most eruption conditions are in the regime of brittle-like fragmentation. Second, under brittle-like fragmentation conditions, a single decompression triggers successive and sustained explosions similar to Plinian-type continuous explosive eruptions^31^. Third, whether gas liberation occurs in a ductile or brittle process may be a controlling factor of the fragmentation speed, as hypothesized by Alidibirov^17^.

In clarifying the mechanism of brittle-like fragmentation, it is crucial to make a one-to-one link between the rupture initiation point and the internal structure of the specimen. The use of X-ray microtomography has expanded to the field of experimental volcanology^32–34^. In this study, the three-dimensional bubble structures of specimens before decompression were characterized by X-ray microtomography, and the dynamic response of the specimens during rapid decompression were recorded using high-speed photography with visible light illumination. A connection was drawn between the crack opening on the specimen surface and the internal bubble distribution. The evolution of stress and the deformation field of each specimen due to decompression were also numerically computed using the finite element method (FEM). A linear Maxwell model was employed to describe the viscoelastic properties of the specimen. Based on the obtained results, the effects of the size and spatial distributions on the development of cracks and the triggering of brittle-like fragmentation are discussed.

## Methods

### Specimen

Following our previous research^12,13^, syrup containing bubbles was used as a magma analog. Maltose syrup (Maltrup, Hayashibara) was used as the specimen material. Syrup is a suitable material for simulating the fragmentation of vesicular magma, because it has a large rigidity *G* (=1 GPa) close to that of magma^30^ and can have a wide range of viscosities *η* from 10^2^ to 10^9^ Pa⋅s. The mechanical properties of the syrup have been summarized in our previous paper^35^. Hydrogen peroxide solution (30%) was mixed as an additive to generate the bubbles in the syrup. Manganese dioxide powder was also added as a catalyst of decomposition of hydrogen peroxide solution.

### Rapid decompression test

**Figure 1:**
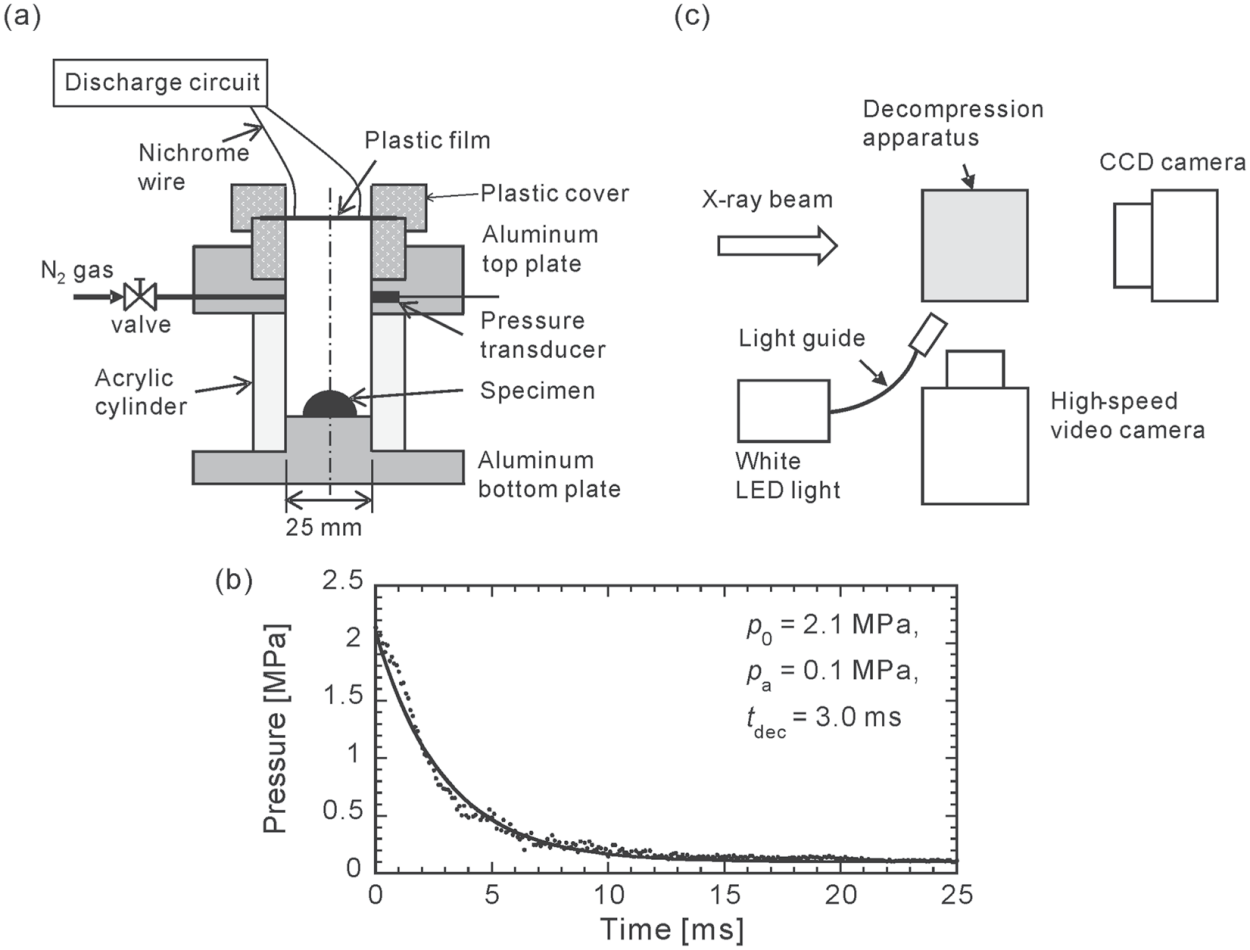
Schematic of the decompression apparatus (**a**) with typical pressure profile (**b**) and imaging system (**c**). Filled circles in (**b**) denotes experimental results. Line in (**b**) indicates approximation of experimental results given by Eq. (1).

A rapid decompression apparatus (Fig. 1(a)) was used to simulate the fragmentation. This apparatus consists of a cylindrical acrylic container with an inner diameter and height of 25 and 40 mm, respectively. The specimen was placed on the aluminum bottom plate of the container. The top of the container was sealed with plastic film. After the specimen was placed in the container, it was pressurized by nitrogen gas via a precision pressure controller (Pace5000, GE). Rapid decompression was applied to the specimen by the quick release of the pressurized gas in the container due to the abrupt rupture of the film. The film was ruptured by Joule heating a thin nichrome wire adhered to the film.

The pressure profile inside the apparatus during decompression is shown in Fig. 1(b). This profile can be approximated fairly accurately with the following exponential decay curve:

1 $$p(t)={p}_{0}\,-({p}_{0}-{p}_{a})\,[1-\exp \,(-\frac{t}{{t}_{{\rm{d}}{\rm{e}}{\rm{c}}}})],$$

where *p*_0_ is the initial pressure just before decompression, *p*_*a*_ is the ambient atmospheric pressure, and *t*_dec_ is the characteristic decompression time. In this experiment, the initial pressure *p*_0_ was set to 2.1 MPa, and the characteristic decompression time *t*_dec_ was approximately 3 ms.

Schematic of imaging system is shown in Fig. 1(c). To observe the three-dimensional (3D) internal structure of the specimen, X-ray microtomography experiments were conducted at BL20B2 of SPring-8 in Japan. For the tomographic reconstruction, 1800 projections of the specimen were obtained over a rotation of 180° just before the decompression for each decompression event. For this purpose, the rapid decompression apparatus was rotated by a motorized rotary stage. The projection images were captured by an X-ray image detector consisting of a visible light converter (BM5, Hamamatsu Photonics) and a 16-bit scientific-grade complementary metal–oxide–semiconductor (sCMOS) camera (C11440-22C, Hamamatsu Photonics). Each projection image has a size of 2048 × 1200 pixels with an effective pixel size of 15.5 *μ*m/pixel, enabling an image of the entire specimen to be obtained. The exposure time of each projection was 100 ms, and the total acquisition time was approximately 180 s.

The dynamic behavior of the specimen during decompression was then observed using a 12-bit digital high-speed video camera (Phantom V710, Vision Research) with a white light emitting diode (LED) as illumination. The framing conditions of the high-speed video camera were a frame rate of 10,000 frames per second (fps), an image size of 512 × 384 pixels, and a spatial resolution of 0.10 mm/pixel.

The pressure profile during decompression in the container was measured using a semiconductor pressure transducer (XTM-190-2500SG, Kulite). The measured signal was amplified through a direct current (DC) amplifier (SA-59, TEAC) and stored in a 16-bit digital data logger (NR-600, Keyence) with a sampling rate of 10 kHz.

### Image processing

A volumetric 3D model of the specimen was constructed from the X-ray transmission images using the following procedure. First, a tomographic grayscale image of each horizontal slice (2048 × 2048 pixels) was reconstructed using the convolution back-projection (CBP) method, the source code of which is open to the public on the SPring-8 website^36^. Next, image segmentation was conducted using code written in Matlab that employed a compilation of standard techniques^37^, including basic global thresholding to generate a binary image, global filtering using a median filter, erosion and dilatation to further reduce salt-and-pepper noise and close any pores, and edge-linking to extract the outer shape of the specimen. Finally, three-dimensional voxel data construction; labeling of the bubbles; and determination of the bubble volume, centroid, and mean void fraction of the specimen were conducted using a commercial 3D image processing software (Simpleware, Synopsys).

### Numerical simulation

The FEM was applied to compute the time-dependent stress and deformation fields inside the specimen due to the overpressure of bubbles after decompression. COMSOL Multiphysics ver. 5.2a was used as the calculation platform. The specimen material was assumed to be a Maxwell fluid with mechanical properties equal to those of the syrup: a rigidity of *G* = 1 GPa, a zero-shear viscosity of *η* = 50 MPa⋅s, and a bulk modulus of *K* = 8 GPa. To reduce the computational cost, the 3D structure of the specimen was simplified in the simulation: a few bubbles that may have triggered the fracture were selected for inclusion in the model, and all other bubbles were removed. The computational grid was generated using the same software that was used for the image processing (Simpleware, Synopsys). The number of elements was 400,000, and the minimum spacing was fixed at 6 *μ*m.

## Results

### Observation

The responses of four specimens are presented here. The experimental conditions for the four considered cases (cases A–D) are listed in Table 1 where *t*_f_ denotes the onset of fragmentation after the decompression was started, and *d*_max_ and *d*(vol.) are the equivalent diameter of the largest bubble and the volume mean diameter, respectively. The relaxation time of a Maxwell fluid is *τ*_r_ = *η*/*G*, and the characteristic time of the viscous expansion of bubbles is *τ*_v_ = 4*η*(1 − *ϕ*_0_)/(3*p*_0_)^13,25^. In this experiment, hemispherical specimens were prepared with a diameter of 20 mm and a height of 10 mm. The viscosity *η* of each specimen was set to a fixed value of 50 MPa⋅s at room temperature (20 °C). The mean void fraction *ϕ*_0_ before decompression is changed from 3% to 35%, and the bubble structure spontaneously varied. As listed in Table 1, all four of the cases have nearly equal sets of characteristic times, and the decompression times *t*_dec_ are much shorter than the relaxation time *τ*_r_ as well as the characteristic time of the viscous expansion of bubbles *τ*_v_. Thus, the specimens were expected to show a solid-like response.

**Table 1:** Summary of experimental conditions.

case	*ϕ*_0_ %	*t*_dec_ ms	*t*_f_ ms	*τ*_r_ ms	*τ*_v_ s	number of bubbles	*d*(vol.)mm	*d*_max_mm
A	34.3	3.3	1.6	50	20.8	9537	5.39	6.49
B	7.6	3.0	2.2	50	29.3	5180	2.78	3.43
C	5.3	2.7	497.0	50	30.0	4858	1.62	2.49
D	3.8	3.5	—	50	30.6	9570	0.58	1.30

**Figure 2:**
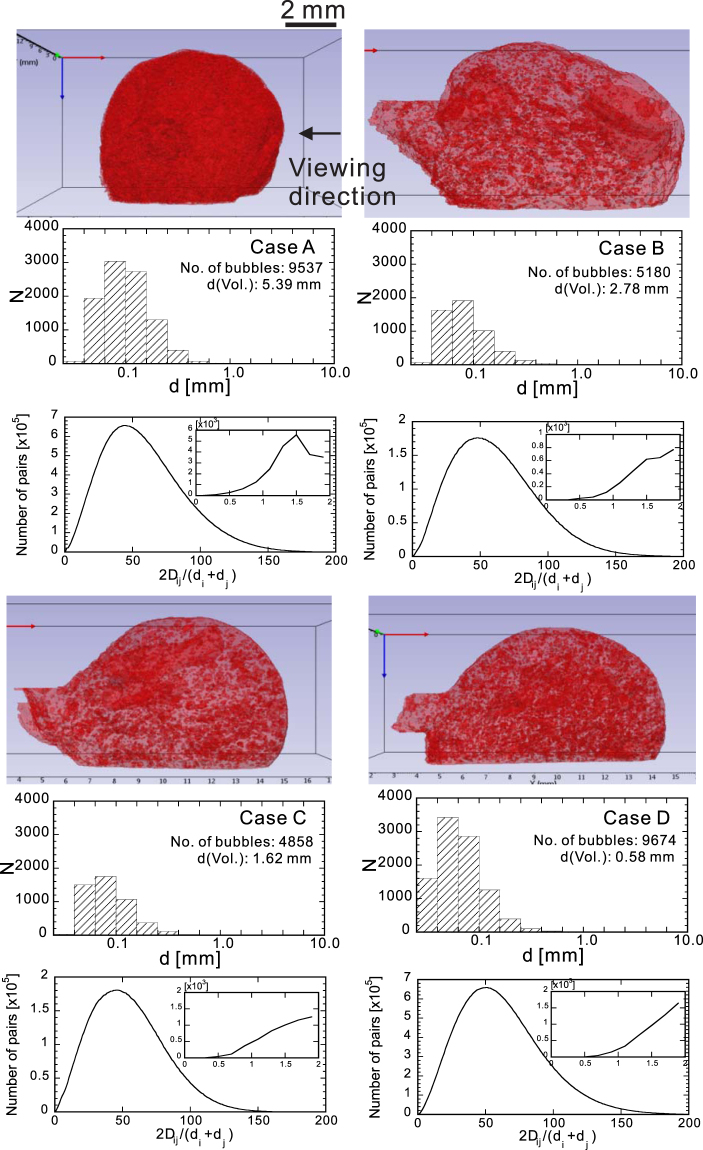
Reconstructed 3D images of specimens with the histgrams of the bubble size and the inter-distance of all the pairs of bubbles. The arrow indicates the viewing direction of the high-speed camera. The total number of bubbles identified in the 3D images is on the order of thousands for each case. The histograms of bubble size indicate that the mode is approximately 0.1 mm, which is much less than the volume mean diameter *d*(vol.). This means that each specimen contains a few large primary bubbles.

Reconstructed three-dimensional images of the specimens are shown in Fig. 2 along with the size and inter-distance distribution histograms of the bubbles. In case A, the bubbles are densely distributed in whole region of the specimen. In cases B and C, a few primary large bubbles exist beneath the outer surface of the specimen. The two primary bubbles in Case B have the equivalent diameter *d* of 3.4 mm and 3.2 mm, respectively. In case C, a tube-shaped bubble whose equivalent diameter is 2.5 mm is placed close to the top end of the specimen. In case D, remarkable primary large bubbles are not found in the specimen. The inter-distance between a pair of bubbles (*i* and *j*) is measured by the distance between their centroids, *D*_*ij*_, normalized by their mean diameter [= (*d*_*i*_ + *d*_*j*_)/2]. The statistics of the inter-distances of all the bubble pairs are similar irrespective of cases at a glance, because they depend primarily on the size of the specimen and the mode of bubble diameter. As shown in the zoomed-in inset, it is noted that several thousand pairs of bubbles have normalized inter-distance of less than 2, which means the film thickness between such a bubble pair is expected to be the order of their bubble diameters.

**Figure 3:**
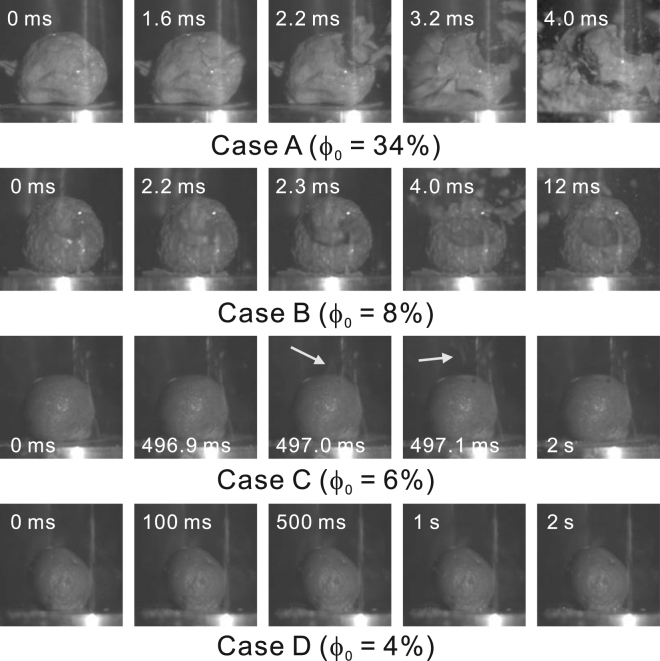
Responses of specimens. Snapshots were taken using a high-speed video camera. The times shown in the individual pictures are the elapsed times after the start of decompression. In case A, a visible crack appeared at 1.6 ms, then partial fracture occurred at 2.2 ms, and specimen burst at 3.2 ms. In case B, partial fracture occurred at 2.2 ms, and subsequent fragmentation events were not observed. In case C, partial fracture was substantially delayed and only occurred at 497 ms. This delay is much greater than the relaxation time *τ*_r_ (= 50 ms). White arrows indicates the flying fragments. Finally, in case D, no visible change was observed during recording.

**Figure 4:**
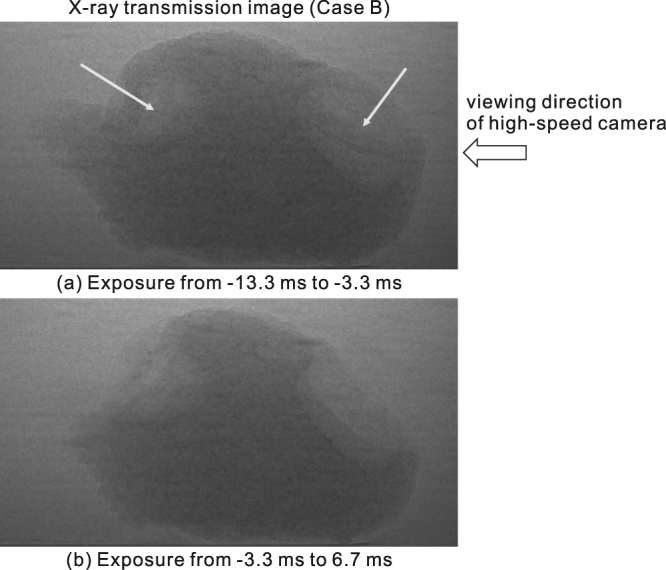
Time-resolved transmission images by X-ray radiography which were photographed from a transverse direction to the high-speed camera for Case B. These images were taken under 100 fps with the shutter left open. The part of specimen around the two large bubbles (pointed by arrows) disappeared within 6.7 ms after the decompression was started.

Images taken by high-speed photography depicting the responses of the porous specimens are shown in Fig. 3. Additionally, the time-resolved transmission images by X-ray radiography which were photographed from a transverse direction to the high-speed camera is shown in Fig. 4 for Case B. Fragmentation may be considered to occur more efficiently when the specimen has a larger void fraction. This tendency generally agrees with the results of previous fragmentation experiments on pyroclastic rock samples^3^, in which the pressure threshold for complete fragmentation was found to be inversely proportional to the void fraction.

There are three problems to be considered. First, the specimens in cases B and C, which have similar void fractions (Table 1) and bubble structures (Fig. 2), showed quite different degrees of fragmentation. Second, the specimen in case C underwent brittle-like fragmentation despite the experimental conditions being in the regime of brittle fragmentation according to the models developed in previous studies^12,13^. Third, the mechanism of the time delay is unknown. The first problem was investigated numerically, and the results are given in the next subsection. The second and third problems are discussed in Discussion.

### Stress field

**Figure 5:**
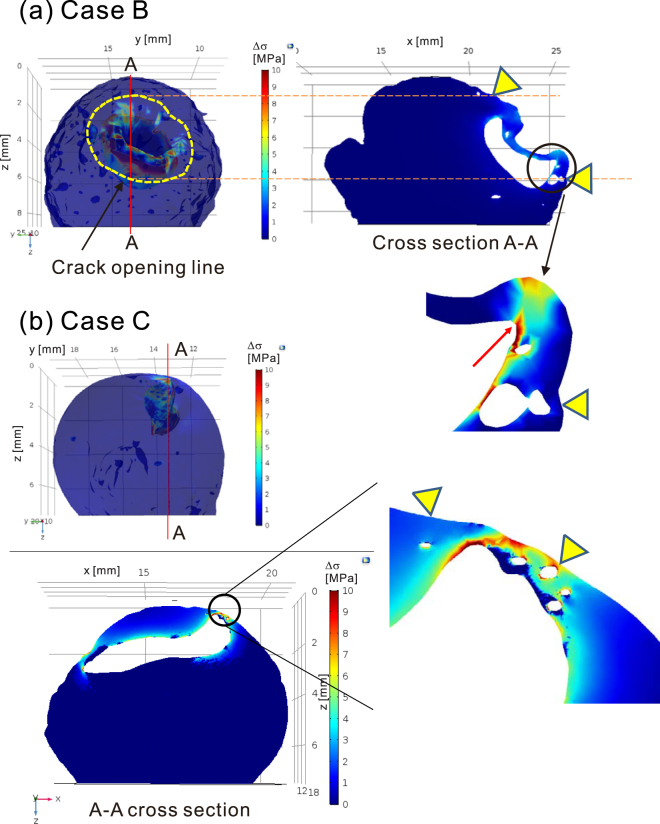
Computed differential stress Δ*σ* field inside the specimens. (**a**) Case B. The dashed yellow line and triangles indicate the location of the surface crack observed by high-speed photography. (**b**) Case C. The triangles indicate the location of the surface crack observed by high-speed photography.

This subsection presents the numerical calculation results for the stress field in the specimens around the location of the initial surface rupture in cases B and C. The surface profiles and cross sections of the model used in the calculation are shown in Fig. 5. Both specimens were found to have a primary large bubble close to the location of the initial surface rupture. As explained in the Methods, only the primary bubble and the small bubbles close to the rupture point were included in the model. Figure 5 shows the locations of the crack openings observed by high-speed photography and a map of the magnitude of principal stress difference (differential stress).

In case B (Fig. 5(a)), the primary bubble was located beneath the dented surface of the specimen, and a circular fracture formed around the dent. Additionally, a few small bubbles near the primary bubble, hereafter called satellite bubbles, were located just below the line of the crack. The differential stress was concentrated in the area extending from the surfaces of the satellite bubbles to that of the primary bubble. Interestingly, even though the differential stress is also concentrated along the rim of the dent, as indicated by a red arrow in Fig. 5(a), the line of crack did not pass through this region. In case C (Fig. 5(b)), a satellite bubble was located near the line of the crack. However, the stress was concentrated only at the surfaces of the satellite bubbles and was low near the primary bubble. The thicknesses of the regions between the primary bubble and the satellite bubbles were larger in case C than in case B.

**Figure 6:**
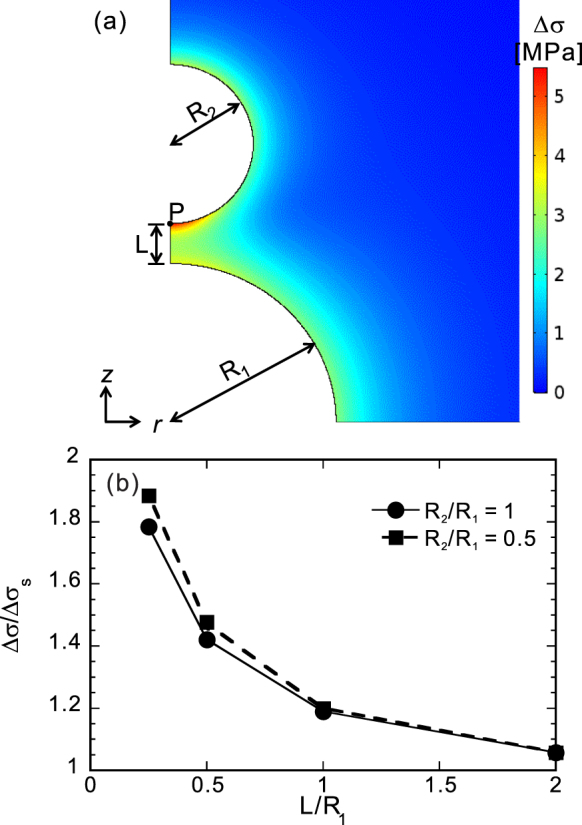
Mutual interaction of differential stress fields in two bubbles. (**a**) Close-up snapshot of the differential stress field at *t* = 30 ms. (**b**) Differential stress at point P under various distances between the two bubbles. Note that the structure is axisymmetric with respect to the left border of the figure, and plane symmetric with respect to the bottom border.

To provide a clear description of the mutual interaction among neighboring bubbles, the stress field around two bubbles was calculated in the axisymmetric domain. The calculation area was a large sphere with a radius of 10 mm. The first bubble (radius *R*_1_ of 1 mm) was located at the center of the calculation domain, whereas the second bubble (radius *R*_2_ of 1 or 0.5 mm) was located near the first bubble along the axis of symmetry. Figure 6(a) shows part of the calculated area around the bubbles. The mechanical properties of the viscoelastic body and the profile of decompression were the same as in the laboratory experiment. The internal pressure of bubbles was assumed to remain constant because the calculation time was shorter than the characteristic bubble expansion time *τ*_v_.

The stress field was calculated at various distances *L* between the surfaces of two bubbles. Figure 6(b) shows the differential stress at point P normalized by the hoop stress around an equivalent spherical shell Δ*σ*_*s*_^18,25^, which is given by

2 $${\rm{\Delta }}{\sigma }_{{\rm{s}}}(r)=\frac{3}{2(1-\varphi )}\frac{{a}^{3}}{{r}^{3}}({p}_{{\rm{i}}{\rm{n}}}-{p}_{{\rm{o}}{\rm{u}}{\rm{t}}})\quad (\,r\,\ge \,a),$$

where *r* is the distance from the center of the shell, *ϕ* is the porosity of the shell, *a* is the bubble radius and *p*_in_ and *p*_out_ are the pressures inside the bubble and outside of the shell, respectively. In the case of decompression, *p*_in_ > *p*_out_ is satisfied. The hoop stress at *r* = *a* with *ϕ* = 0 was used to normalize the differential stress, because the porosity *ϕ* is negligible (approximately 0.1%) in this calculation.

As shown in Fig. 6(a), the differential stress is concentrated at point P. The stress appears to become concentrated at the surface of a bubble when another bubble is present nearby. Interestingly, the differential stress increases as the radius of the nearby bubble decreases. Figure 6(b) indicates that the differential stress at P is significantly larger than the hoop stress of an isolated bubble if the distance *L* is less than the bubble radius *R*_1_. Figure 6(b) suggests that a large primary bubble has a larger influence on the stress field around small satellite bubbles.

These results confirm the important effect of bubble distribution on the stress field and thus on the initiation of rupture. In case B, the rupture penetrated the primary bubble. A considerable amount of gas was released from a crack, which triggered fragmentation, as has been reported by Kameda *et al*.^13^. However, in case C, the rupture occurred only at subsurface satellite bubbles and did not have a sufficiently large impact to trigger fragmentation.

## Discussion

Two remaining problems and a new scenario for the brittle-like fragmentation are discussed in this section.

First, the condition for brittle-like fragmentation proposed in our previous studies^12,13^ must be reconsidered, because the fragmentation was observed when *t*_dec_ was much shorter than *τ*_r_.

Heap *et al*.^19^ proposed a different condition for the fracture and fragmentation of magma based on their numerical simulation of an elastic solid containing overpressure bubbles of a uniform size. The nucleation and propagation of a fracture are mainly controlled by the bubble distribution. The fragmentation triggered by the fracture is sustained if the stress criterion around the bubbles is satisfied. The present results are consistent with this condition but with the addition of the effect of the bubble size distribution on the onset of fracture. The specimens used in the present study have diameters of approximately 20 mm, whereas those used in our previous experiments had diameters exceeding 50 mm^12,13^. In a larger specimen, bubble structure that facilitates fractures are more likely to exist. Therefore, fragmentation is triggered more efficiently in a larger specimen. Brittle and brittle-like fragmentation may not be distinct types of fragmentation that occur in exclusively distinct conditions; instead, the fragmentation type may depend on a continuously and probabilistically changing fragmentation threshold.

The variability in the effect of bubble interactions is expected to be larger for smaller specimens and specimens with an intermediate void fraction. A stress concentration is expected when the surface of a bubble is located within stress influence of the neighbor bubble, which Eq. (2) suggests to be within twice of the bubble radius (*r*/*a* < 2). Notably, the porosity of the spherical shell is *ϕ* = (*a*/*b*)^3^, where *b* is the outer radius of the shell. If the diameters of all the bubbles and the distances between them are equal, the distance between the surfaces of a pair of adjacent bubbles is equal to 2(*b* − *a*). Therefore, the stress concentration induced by bubble interaction is remarkable when the distance 2(*b* − *a*) is smaller than the bubble radius *a*, which corresponds to the porosity $\varphi ={(a/b)}^{3} > {(2/3)}^{3}\simeq 0.3$ (30%). Bubble interactions will be rare if the void fraction is very small. This hypothesis is supported by the fragmentation threshold for pyroclastic rocks by a shock-tube experiment^3^ which varies widely for specimens with intermediate porosities ranging from 10% to 20%.

Second, what controls the delay in the onset of fracture is an unresolved issue. It was inferred in this study that a slow but irreversible crack grows as a result of the stress concentration.

Shahidzadeh-Bonn *et al*.^38^ reached a similar conclusion from their experiment on the fracture of porous glass at room temperature. They observed that the delay in the onset of fracture varied in a range spanning more than three orders of magnitude (from less than one second to thousands of seconds) even if they used specimens with equal bulk structural and mechanical properties. They concluded that the delay is a consequence of the nucleation of the primary critical crack. The existence of unhealed microcracks leads to weaker regions, which accelerates the failure.

However, the existence of microcracks in the present viscoelastic fluid specimens is not realistic. Instead, it was considered here that during the delay time, ductile deformations proceeded in some parts of the specimen. Such deformations proceed on a shorter time scale than *τ*_v_ because of the localized large stress, and they may accelerate the increase in the stress concentration. The material response becomes brittle when the rate of stress increase is sufficiently large^39^, and finally brittle fracture occurs. This scenario will be confirmed by fracture mechanics simulation for a Maxwell fluid in future work.

Finally, we propose a new scenario for the brittle-like fragmentation in magma flowing upward in a volcanic conduit on the basis of this study.

Existing fragmentation models are based on shock tube-type models and experiments and have been applied to actual volcanic fragmentation triggered by rapid decompression, such as dome collapse and plug breakage. The fragmentation process in the expansion flow of magma in a continuous eruption has not yet been well identified. Although a critical strain rate criterion has been proposed^40^, it has been demonstrated that if the shear thinning nature is considered, an increasing strain rate does not necessarily increase the brittleness of the flowing magma^39^.

In this study, it was found that inhomogeneous spatial and size distributions of bubbles are essential causes of the initial fracture that triggers delayed fragmentation. This indicates that the history of bubble nucleation, growth, and coalescence in magma control the fragmentation behavior of the magma. In other words, both the instantaneous decompression rate and the history of decompression are important in determining the fragmentation behavior. The present experimental and numerical results have demonstrated that fracture is efficiently initiated when small bubbles are located in the vicinity of a large bubble. The second nucleation of bubbles, which is considered to occur near the fragmentation surface^41–43^, will generate such bubble structures.

**Figure 7:**
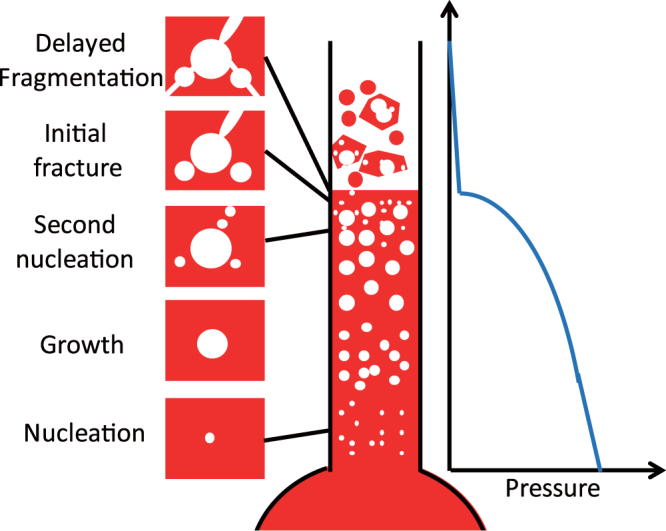
Proposed scenario of brittle-like fragmentation in magma flowing upward in a volcanic conduit.

A sketch of the new scenario for the brittle-like fragmentation is shown in Fig. 7. The velocity and decompression rate of the upward flow in the conduit increase toward the fragmentation surface^40^. However, the decompression rate remains too small to cause brittle fragmentation. Instead, the second nucleation occurs, generating small bubbles around the large preexisting bubbles, which have been termed pheno-bubbles by Toramaru^43^. The stress is concentrated in some regions between the pheno-bubbles and secondary bubbles, and it increases further at large stresses as a result of nonelastic deformation. The local rate of stress increase then becomes sufficiently large to initiate crack propagation. If the magma has the bubble structure that helps cracks to reach the magma surface, the gas in the crack is rapidly released. Then, delayed fragmentation occurs, as was observed in our previous laboratory experiments^13^.

In order to justify this scenario in actual eruptions, we need in the future to relate the fragmentation mechanism to information measurable from pyroclastic deposits such as internal structures (size and spatial distributions of bubbles, vesicularity, and permeability) and entire properties (total grain size distribution)^44–46^.

In conclusion, the occurrence of fragmentation is significantly influenced by the internal structure of the sample. Fragmentation tends to occur when the spatial and size distributions of bubbles in the sample are nonuniform. Numerical analysis revealed that when a small bubble is located close to a large bubble (within a distance equal to twice the radius of the large bubble), significant stress amplification occurs in the region between the two bubbles. In the experiments and imaging, crack opening and subsequent fragmentation were observed to start at such structures. Based on these results, a new fragmentation mechanism was proposed. In the proposed scenario, the second nucleation of bubbles near the fragmentation surface is essential for the advancement of brittle-like fragmentation in an upward magma flow in a volcanic conduit.
